# Web-Based Depression Screening and Psychiatric Consultation for College Students: A Feasibility and Acceptability Study

**DOI:** 10.1155/2014/580786

**Published:** 2014-03-30

**Authors:** Aya Williams, Rachel LaRocca, Trina Chang, Nhi-Ha Trinh, Maurizio Fava, Joseph Kvedar, Albert Yeung

**Affiliations:** ^1^Massachusetts General Hospital Depression Clinical and Research Program, One Bowdoin Square 6th floor, Boston, MA 02114, USA; ^2^Massachusetts General Hospital Center for Connected Health, 25 New Chardon Street, Suite 300, Boston, MA 02114, USA

## Abstract

*Background*. A steady rise in the prevalence of depression among college students has negatively affected student quality of life. This study investigates the feasibility and acceptability of a Web-based model, including Skype, to screen and provide psychiatric consultation to depressed college students. *Methods*. Students completed the 9-item Patient Health Questionnaire (PHQ-9) online; those who screened positive (PHQ-9 ≥ 10) or endorsed any level of suicidal ideation were offered Web-based psychiatric consultation using Skype. After the consultation, students filled out a 7-item satisfaction questionnaire to report on the acceptability of this Web-based method. *Results*. A total of 972 students consented to the online depression screening and 285 screened positive. Of those, 69 students consented and 17 students successfully completed the psychiatric consultation via Skype. Thirteen (76.4%) students found the interview useful in helping them understand their depression. Fifteen (88.2%) students thought that psychologists and psychiatrists could successfully see patients via videoconferencing. *Conclusions*. Current online technologies can provide depression screening and psychiatric consultation to college students; those who participated reported a positive experience. Future studies will need to address the low levels of participation among college students and attract students who are underserved, as well as use a videoconferencing platform that adequately protects data confidentiality.

## 1. Introduction

Major depressive disorder (MDD) is a highly prevalent illness affecting 2% to 3% of men and 5% to 9% of women in the United States at any particular point in time, with a lifetime risk of 5% to 12% in men and 10% to 25% in women [1]. A steady rise in the prevalence of depression among college students [2, 3] has negatively affected students' quality of life. According to the 2007 national survey across 39 university campuses, 43.2% of students reported “feeling so depressed it was difficult to function” and 10.3% admitted to “seriously considering attempting suicide” in the past year [4]. MDD among college students is associated with diminished academic performance [5, 6], increased alcohol consumption [7], increased maladaptive eating [8], compromised physical health [9], and increased suicidal ideation [10]. A depression diagnosis made during college is associated with twice the increased risk of discontinuation of the student's college career [11]. A systematic review of depression prevalence studies in university students suggests that these figures may underestimate the actual prevalence [12]. Thus, identification and management of depression in university settings are necessary.

Several studies have used Web-based surveys of depression and anxiety [13] to screen successfully for mood problems among college students [10, 14, 15]. However, online screening alone does not connect students with necessary resources for treatment. Up to 85% of students with positive screens for mental disorders do not receive any treatment from professionals [16]. Reasons include lack of problem recognition and need, delayed time to appointment, lack of time, financial constraints, and privacy concerns [14]. There is a need to address these barriers that exist between receiving a positive screen result and accessing professional treatment. Kim et al. [13] argue that screening must be integrated with accessing professional services, so when a mental health need is identified via screening, a student can act on the need in the same session. Hass et al. [17] suggested that clinician-student dialogue and personalized assessment, unlike computer-generated feedback, could identify and help students resolve their resistance to treatment. Mailey et al. [18] similarly emphasized the need for a more interactive model of Internet interventions with live clinician instead of text-heavy communications to keep students socially engaged. A review of technology-assisted self-help and minimal contact therapies confirmed that therapist-assisted treatments remain optimal in the treatment of clinical depression [19].

Given these findings, researchers and clinicians have turned to videoconferencing, which allows real-time interaction between the student and the clinician while obviating the cost of logistical barriers such as transportation and the potential stigma surrounding visiting an on-campus mental health clinic. The clinician can gauge the student's appearance, including the choice of clothing and attire, facial expression, and bodily movement and use these visual cues to inform clinical diagnosis and treatment recommendations [20]. Many online counseling services have recently emerged [21–23]. Yet there are few studies assessing the effectiveness and safety of such therapies for mental health in the university setting [24]. Khasanshina et al. [25] developed a model of psychiatric services that connected students experiencing high distress with clinicians via videoconferencing. The model successfully supplemented mental health care for college students in the rural university setting, although the services could be delivered only at a clinic connected by a virtual private network.

Studies have examined the feasibility and acceptability of Skype to deliver psychotherapy to clinical populations, with positive results [26–28]. In a study measuring the feasibility and efficacy of exposure therapy for social anxiety disorder using Skype, 83% of the therapists rated delivering treatment through Skype as fairly or very feasible, and 95% of the patients reported that receiving treatment through Skype was fairly or very easy [26]. Two studies investigating the efficacy of cognitive behavioral therapy [27] and problem-solving therapy [28] through Skype for patients with depression demonstrated clinically significant improvement in depressive symptoms. Technical difficulties, such as sound quality (e.g., choppiness, softness, echoing, and delay) and video quality (e.g., blurriness, frozen picture, and delay), still remained in these studies. Skype was run on desktop computers or laptops from home or primary care offices. Moreover, telephone screening or in-person setup of the technology occurred prior to the initiation of Skype psychotherapy.

This study investigated the feasibility and acceptability of a Web-based model that reached out to college students for online depression screening immediately followed by scheduling of an online psychiatric consultation if the student screened positive. The entire procedure from recruitment to consultation was carried out online, with no in-person screening or setup. The consultations were offered by licensed off-campus psychiatrists using Skype, a free videoconferencing software. The study replicated a real-world setting in which students used their own portable laptops with built-in webcams over wireless networks on campus. The quality of consultations was measured using a self-rated satisfaction questionnaire. We hypothesized that this Web-based model to enhance recognition and treatment of depressed college students is both feasible and acceptable.

## 2. Materials and Methods

### 2.1. Screening and Recruitment

Undergraduate and graduate students currently enrolled in universities in Massachusetts were recruited to participate in the Web-based screening survey for depression between December 2010 and December 2011 through Facebook (http://www.facebook.com/), Craigslist (http://craigslist.org/), and paper and electronic school flyers. Approximately 1,500 flyers were placed in student resident mailboxes at one local university in Boston. Students who participated in the study were entered in a raffle and had a 1 in 15 chance of winning a $20 gift card. All subjects provided informed, voluntary, written consent. The study and the use of Skype for a clinical consultation were approved by the Institutional Review Board.

The screening survey collected information on student demographics, including sex, year in college, and past and current history of depression and treatment, as well as the 9-item Patient Health Questionnaire (PHQ-9) for depression screening. A student was considered screen-positive for MDD if he or she scored 10 or above on the PHQ-9 or scored 1 or above on the PHQ-9 suicide item (question 9). If a student indicated any suicidality, the survey prompted the student to visit the nearest emergency room or call 911 before proceeding in the survey. If a student scored less than 10 on the PHQ-9 and did not endorse suicidality on the PHQ-9, he or she was identified as screening negative for MDD.

All students who completed the depression screening questionnaire received a direct link to an online depression toolkit at the conclusion of the survey regardless of their PHQ-9 score. The toolkit included links to two websites providing psychoeducational materials on depression, as well as information on a local suicide prevention helpline.

Participants who screened positive for MDD or suicidality were given an additional opportunity to schedule a Skype consultation with one of three research psychiatrists using “Doodle Meet Me” (http://www.doodle.com/about/meetMe.html), which showed clinicians' schedule availability in the following two weeks. To increase transparency, the final survey page included an explanation of the Skype consultation, as well as a direct hyperlink to the principal investigator's biography that provided detailed information of the clinician the students would speak with.

### 2.2. Web-Based Consultation

Measures were taken in this study to meet the Practice Guidelines for Video-Based Online Mental Health Services [29] of the American Telemedicine Association. Students provided a second informed, voluntary, written consent when scheduling the Web-based consultation. Prior to the videoconferencing session, a clinical research staff member verified the contact information of the student including a nonidentifying username, e-mail address, and phone number. The research staff member subsequently completed an online trial conversation with the student to test the connectivity and the functionality of the clinic's videoconferencing system. The online consultants are licensed psychiatrists who are experts in depression. The senior author (Albert Yeung) was responsible for most of the consultations. He has extensive experience in Skype-based clinical interviews and has performed more than 100 online consultations.

At the beginning of the one-hour Web-based consultation using Skype, the clinicians introduced themselves and identified the names of the students by asking them to present their school student identification card with a photo and the location of the student. Clinicians completed the sessions from their outpatient clinic offices and students were on their respective university campuses. The clinicians ensured that the participant was in a private room where the clinical discussion would not be easily overheard by others. The student used his or her own personal computer and webcam.

The consultation used a standardized clinical review of the history of present illness (including psychiatric, medical, psychosocial, and family history) and the mental status exam to generate the psychiatric diagnoses according to the Diagnostic and Statistical Manual of Mental Disorders (DSM-IV-TR), followed by a discussion of available options for treatment. In addition, the clinician solicited the student's feedback on the use of online screening and the consultation service. The details of the clinical interview were recorded and securely stored. At the conclusion of the consultation, clinicians sent a consultation report to the students' care providers if students signed a statement to release their medical information and made referrals as necessary.

All students who completed the one-hour Web-based consultation received a Skype satisfaction questionnaire within a week to inquire about the sound and visual of the Web-based interview, its helpfulness in understanding depression and starting treatment, and their opinions about the use of Web-based videoconferencing for mental health services.

Eight weeks after the completion of the initial survey, students who consented to be recontacted were e-mailed follow-up surveys, which included a second PHQ-9 questionnaire, questions about the use of online resources provided, any treatment sought by the student after the completion of initial survey, reasons for not seeking treatment, and obstacles to seeking treatment for depression. Again, students who responded were entered into a raffle with a 1 in 15 chance of winning a $20 gift card.

### 2.3. Instruments for Online Depression Screening

The 9-item Patient Health Questionnaire is a self-administered instrument that can be used to screen for patients with MDD in primary care settings. Each item in the questionnaire corresponds to one of the nine criteria upon which the DSM-IV-TR major depressive diagnosis is based. In a validation study of the PHQ-9, Kroenke and colleagues [30] showed that the instrument had a sensitivity of 88% and specificity of 88% for identifying MDD when using a total score of 10 or above as the threshold.

### 2.4. Software for Online Videoconferencing

Skype is a voice-over-Internet protocol service and software application that allows users to communicate by voice, video, and instant messaging over the Internet. Skype counts more than 300 million registered users [31] and is currently the most used desktop videoconferencing application [32]. The application can be downloaded free of charge on multiple platforms, including personal computers, tablets, and smart phones. Skype employs peer-to-peer communications, which means that computers exchange messages directly, without going through a third party server [33]. The students were asked to create a new Skype username that did not include identifying information such as birthday or name. The clinician also used the general clinic Skype account. Skype meets Advanced Encryption Standards, which meets the Federal Information Processing Standards for electronic transmission under HIPAA [34, 35].

## 3. Results

A total of 972 students consented to participate in this study. The majority of the respondents were female (71%) and Caucasian (67%). Freshmen (25%) and sophomores (24%) each made up a quarter of the total respondents, followed by juniors (17%) and seniors (17%) (Table 1). The screening survey was largely successful; approximately 10% of the students did not fully complete the assessment. Of the 861 students who completed the survey, 262 students (30.4%) screened positive for MDD based on a PHQ-9 score of 10 or above, and 151 students (17.5%) endorsed suicidal ideation on item number 9 of the PHQ-9.

**Figure 1:**
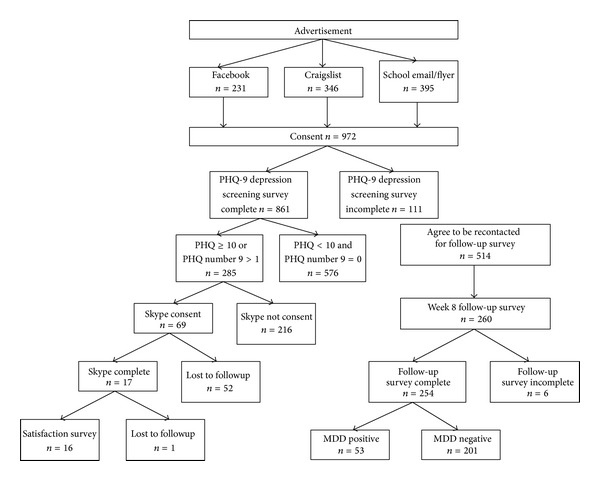
Subject participation and study flow.

Among those (*n* = 285) who screened positive for depression or had a positive response to the suicidality item of PHQ-9, 69 (24%) consented to participate in the Web-based psychiatric consultation and 17 (6% of eligible participants) successfully scheduled and completed the Skype consultation (Figure 1). The majority of students reported that the sound (87.5%) and video (81.3%) quality of the consultation were clear. Most students (81.3%) found the interview useful in understanding their depressive symptoms. Fewer students (18.8%) found the interview useful in starting treatment for depression. Overall, completers provided overwhelmingly positive feedback (93.8%) on the likelihood that psychologists and psychiatrists could successfully see patients via Web-based consultation service (Table 2).

Qualitative comments from the students also support the idea that Web-based consultation is an “effective” and “successful” alternative to an in-person clinic visit. One student commented, “It is relatively easy for me to go to a nearby hospital. But I know some of my friends who would have a much harder time doing that, so I would definitely recommend Skype as an appropriate substitute.” Another student recommended the service especially for those who “may not have the capability of leaving their houses.” Students identified the benefit of being “able to connect with a doctor from my apartment where I was comfortable.” One student commented that with the use of Web consultation, “I may have been able to start my treatment earlier.” Students moreover noted the preference for visual cues, stating “body language and seeing someone interact in one-on-one situation is very important.” With the use of the webcam from the students' computers, the consulting clinicians were not able to adjust webcams remotely during the interview. Yet, the consulting clinicians felt that this did not affect their ability to make psychiatric diagnosis and consult the students (personal communications with N.T., T.C., and A.Y.).

A total of 254 students completed the eight-week follow-up survey. According to the second PHQ-9, 53 students (20.9%) scored positive for depression. Forty-three students (16.9%) sought treatment for depression since completing the initial screening survey. Fifteen students (6%) used online resources including the depression toolkit. A total of 123 students (51.5%) indicated “too busy/I forgot” as the reason for not using online resources in the eight weeks following the screen. Thirty-three students (56.9%) indicated that they “did not experience any obstacles” when seeking depression treatment.

## 4. Discussion

This study shows that a comprehensive model of active outreach to college students via online screening, followed by student-initiated online scheduling and a psychiatric consultation using Skype, may be a feasible and acceptable model to improve the recognition and treatment of college students with depression.

This study demonstrates the safety and technical capability for students to use their personal computers and webcams to complete an online depression screening and psychiatric consultation. The use of the Web-based technology eliminated geographical barriers and saved transportation time as well as cost for such screening and consultations. This is of particular importance to college students who have limited time due to numerous academic and social commitments. Moreover, the symptoms of depression can inhibit patients from making the effort to visit a clinic in person. This service offered the option of consulting a professional from the comforts of their own dormitory rooms. A major advantage of the videoconferencing therapy is that it allows for the exchange of visual and nonverbal communications between patients and therapists, unlike telephone or Web-based treatments [26]. These students' qualitative responses supported the use of videoconferencing over audioconferencing, which allowed visual cues such as eye contact, facial expressions, and body language.

Out of the 285 students who screened positive for depression or suicidality symptoms, 69 (24%) showed initial interest in a Skype interview. This finding is consistent with previous studies that found that many college students with significant depressive symptoms do not pursue professional help [36]. It is possible that some of the students who screened positive for depression were “false positives.” They could be experiencing stress-related symptoms during an intense academic semester but not actually have a depressive disorder. However, it is also possible that students may feel comfortable filling out an anonymous survey but still hesitate to discuss their symptoms with a live clinician over a virtual medium. This is a public health challenge that may be related to the stigma regarding psychiatric illnesses [37], the lack of insight into the need for help [38], or concerns about potential administrative sanctions, such as mandatory leave or dismissal from school [17].

These concerns may be alleviated by adding a brief personalized psychoeducation component at the end of the screening material, as well as by providing more information on the content of the psychiatric consultation. An explanation of student's PHQ-9 depression score addressing which specific depressive symptoms the student might be experiencing and their short and long-term negative effects on their life may emphasize the need for professional intervention. A psychiatric consultation with a clinician will offer a more thorough evaluation of this score. The specific details of this consultation, such as what topics are discussed and what treatment options might be available following the session, also might reduce fears about seeing a psychiatrist for the first time. It should be made clear that confidentiality is protected. Informing potential participants of the high acceptability ratings among peers who have received such a consultation may make the service more approachable.

The low rate (25%) of completion of the consultation among interested subjects (17 out of 69 subjects) may be due to the limitation of time and the lack of mental health priority in that limited time. In the eight-week follow-up survey, 123 students (51.4%) indicated “Too busy/I forgot” as the reason for not using online mental health resources. Moreover, 33 students (56.9%) indicated that they “did not experience any obstacles” when seeking treatment for depression. Internal barriers may be playing as important of a role as external barriers to treatment. Yorgason et al. [39] also list the top reason for not using mental health services among college students as their lack of time. Although students were able to immediately schedule their consultation appointments, some students requested appointments one to two weeks after the screening visit, and many were unable to keep them. One session that encompasses both screen and consultation may be advisable [13]. This Web-based model could allow universities to employ outside providers for Web-based consultations or use current providers to practice from off-campus. By increasing clinician time, the service may be made more available during the times when students do not have classes or extracurricular commitments, such as in the early evening.

A more active outreach in the mediating step between the screen and online consultation may increase priority and flexibility of appointment times. In this study, while students were able to schedule an online consultation appointment immediately following the screen, this was a one-time opportunity. Sending automated follow-up e-mails with a link to the online schedule service might increase participation by providing second and third opportunities. This also allows flexibility for college students with changing schedules to move their initial appointment as necessary. It is also possible to allow students to respond to this e-mail or Skype chat with clinical staff to address questions and concerns about the consultation. However, the focus of such a conversation would be to facilitate and encourage the online consultation rather than to address any psychiatric concerns.

Another concern could be raised by the modest number of participants (18.8%) who found the consultation useful for starting depression treatment. About half of the students were already mental health consumers and thus did not need a consultation with a specialist to help them start the treatment for depression. On the other hand, a week may not have been enough time to seek treatment after the consultation. Nonetheless, future studies should be restricted to students who are not receiving any active treatment to focus on depressed college students who are undertreated. A more direct communication between the psychiatrists and the university mental health clinic may be helpful. In this study, the psychiatrist offered consultation report for outside providers. With more concerted efforts between the on-campus clinic and outside providers, the Web-based psychiatric consultation could serve as a gateway to distinguish those students who need further treatment and motivate students' help seeking behavior by sending the report directly to an on-campus clinician.

The 47% of students currently receiving psychiatric treatment who sought this online consultation might suggest a self-selection bias. The experience of previously or currently seeing a clinician in person might have lowered barriers to seek help via online psychiatric consultation. As mentioned earlier, there may be stigma or fear of seeing a mental health clinician for the first time even through a virtual medium. There is a need to make transparent what occurs during the consultation and what options might be available following the session. While currently receiving psychiatric treatment, students screened positive for depression and sought an online consultation. This might indicate the usefulness of this model not only to recognize depression but also to augment an ongoing in-person treatment for students. Overall, the high acceptability rating (93.8%) among those who are currently receiving other psychiatric treatment as well as those who are not indicates the acceptability of this online consultation model.

Finally, there are advantages and disadvantages to using Skype as the platform for an online psychiatric consultation. The advantages of using the software include familiarity and ease of access from computers and multiple types of mobile applications for free [35]. Within the U.S., there are 47 million individuals who already use Skype [31] and do not need to sign up for a new service to speak with mental health professionals. More than half of the users are between the ages of 18–35 [31], which makes this software appropriate for a younger population, such as college students. The disadvantages of Skype involve its security concerns and HIPAA compliance. Skype utilizes 256-bit encryption, which meets the Advanced Encryption Standard specified by the U.S. National Institute of Standard of Technology [34]. Skype operates behind a firewall that can help protect it from unauthorized access [40]. Several experts have noted that it is harder to hack into Skype than into most telephone lines or brick and mortar offices with file cabinets; hacking into Skype requires high expertise and skills [35]. However, there is an ongoing debate as to whether Skype satisfies the HIPPAA Conduit Exception or not [35]. Critics of Skype indicate that Skype does not state on its website that it is HIPAA-compliant and does not offer Business Associate contracts to therapists or clinics that use it for telemental health purposes [35]. Future telepsychiatric consultations should monitor if Skype will address these concerns or should consider using a platform that has demonstrated HIPAA compliance.

## 5. Conclusions

In summary, we achieved some success using a comprehensive Internet-based screening and consultation model to increase recognition and treatment of depressed college students. High acceptability among completers demonstrates that the use of students' personal laptops, built-in webcams, and Skype is a potentially viable model to bring mental health services to college students. The students' qualitative comments suggest the importance of a real-time interaction with a clinician. The low participation rate among eligible students in this study suggests the need for more psychoeducation increase awareness of depression and the benefits of obtaining treatment for depression, as well as a flexible schedule that can adapt to the needs of busy college students. In addition, we recommend that all measures meet the American Telemedicine Association guidelines and that a videoconferencing platform that protects personal confidentiality be used.

This model is valuable for colleges with limited mental health resources on-campus and in their localities, as clinicians who are based at distant sites can now provide their services via Web-based videoconferencing. While we provided a one-time Web-based consultation in this current study, the model could be further developed to provide long-term symptom monitoring and to deliver continued treatment for students with MDD. Future studies on a larger scale and randomized controls are needed to demonstrate this model's efficacy, effectiveness, cost, and potential for expansion to other populations. With rapid increase in the use of webcam-equipped laptops, tablets, and smart phones among both patients and clinicians, this Web-based service may eventually benefit underserved individuals in university settings and beyond.

## Figures and Tables

**Table 1:** Demographics of college students who participated in online depression screening survey and Skype interview.

	Initial screening survey *N* (%)	Skype interview *N* (%)
Consented	972 (100)	17 (100)

Gender		
Male	220 (23)	3 (18)
Female	690 (71)	14 (82)
No response	62 (6)	0 (0)
Ethnicity		
Not Hispanic or Latino	775 (80)	14 (82)
Hispanic or Latino	77 (8)	3 (18)
Do not wish to provide an answer	9 (1)	0 (0)
Not known	111 (11)	0 (0)
Race		
Caucasian	652 (67)	13 (76)
Asian	84 (9)	3 (18)
Black	28 (3)	0 (0)
American Indian or Alaska native	4 (0)	0 (0)
Other	78 (8)	1 (6)
Do not wish to provide an answer	126 (13)	0 (0)
Year in college		
Freshman	242 (25)	7 (41)
Sophomore	230 (24)	4 (24)
Junior	161 (17)	4 (24)
Senior	163 (17)	0 (0)
5th year	43 (4)	1 (6)
Graduate student	41 (4)	0 (0)
Do not wish to provide an answer	92 (9)	1 (6)
Aware or previously diagnosed with depression		
No	456 (47)	1 (6)
Yes	306 (31)	12 (71)
Do not know	110 (11)	4 (24)
Do not wish to provide an answer	100 (10)	0 (0)
Previously received treatment for depression		
No	593 (61)	4 (24)
Yes	271 (28)	13 (76)
Do not know	9 (1)	0 (0)
Do not wish to provide an answer	99 (10)	0 (0)
If yes:		
Medication	56 (21)	2 (15)
Therapy	93 (34)	7 (54)
Both	115 (42)	4 (31)
Do not wish to provide an answer	7 (3)	0 (0)
Currently consider themselves suffering from depression		
No	472 (49)	1 (6)
Yes	171 (18)	8 (47)
Do not know	139 (14)	8 (47)
Do not wish to provide an answer	190 (20)	0 (0)
Currently receiving treatment for depression		
No	629 (65)	9 (53)
Yes	151 (16)	8 (47)
Do not know	4 (0)	0 (0)
Do not wish to provide an answer	188 (19)	0 (0)
If yes:		
Medication	55 (36)	3 (38)
Therapy	28 (19)	3 (38)
Both	61 (40)	2 (25)
Do not wish to provide an answer	7 (5)	0 (0)

**Table 2:** Descriptive statistics of Skype satisfaction survey.

	*N* (%)
Completed	16 (100)

Number 1: audio quality: was the sound clear?	
Clear	14 (87.5)
No opinion	2 (12.5)
Unclear	0 (0)
Number 2: visual quality: was the image clear?	
Clear	13 (81.3)
No opinion	2 (12.5)
Unclear	1 (6.3)
Number 3: was the interview useful in helping you understand your depression?	
Useful	13 (81.3)
No opinion	1 (6.3)
Not useful	2 (12.5)
Number 4: was the interview useful in helping you start treatment for your depression?	
Useful	3 (18.8)
No opinion	9 (56.3)
Not useful	4 (25.0)
Number 5: have you received treatment for depression within two months after the interview?	
Yes	7 (43.8)
No	9 (56.3)
If yes: (*n* = 7)	
Medication	
Therapy	3 (42.9)
Both	4 (57.1)
Number 6: do you think psychologists and/or psychiatrists can successfully see patients via Skype video-conferencing?	
Likely	15 (93.8)
No opinion	0 (0)
Unlikely	1 (6.3)
Number 7: if you were to see a psychiatrist or psychologist, would you prefer to be interviewed face to face or through videoconferencing?	
Face to face	12 (75.0)
Videoconferencing	1 (6.3)
No preference	3 (18.8)
